# *β*-Ketoenamine-linked covalent organic framework for efficient iodine capture

**DOI:** 10.55730/1300-0527.3684

**Published:** 2024-06-15

**Authors:** Onur BÜYÜKÇAKIR

**Affiliations:** Department of Chemistry, İzmir Institute of Technology, İzmir, Turkiye

**Keywords:** Covalent organic frameworks, iodine capture, adsorbent materials, porous organic materials, environmental remediation

## Abstract

Exploring the materials that effectively capture radioactive iodine is crucial in managing nuclear waste produced from nuclear power plants. In this study, a *β*-ketoenamine-linked covalent organic framework (bCOF) is reported as an effective adsorbent to capture iodine from both vapor and solution. The bCOF’s high porosity and heteroatom-rich skeleton offer notable iodine vapor uptake capacity of up to 2.51 g g^−1^ at 75 °C under ambient pressure. Furthermore, after five consecutive adsorption-desorption cycles, the bCOF demonstrates high reusability performance with significant iodine vapor capacity retention. The adsorption mechanism was also investigated using various ex situ structural characterization techniques, and these mechanistic studies revealed the existence of a strong chemical interaction between the bCOF and iodine. The bCOF also showed good iodine uptake performance of up to 512 mg g^−1^ in cyclohexane with high removal efficiencies. The bCOF’s performance in adsorbing iodine from both vapor and solution makes it a promising material to be used as an effective adsorbent in capturing radioactive iodine emissions from nuclear power plants.

## 1. Introduction

Nuclear energy has been considered an alternative energy source to meet growing energy demands and mitigate greenhouse gas emissions [1]. However, managing radioactive nuclear waste from nuclear power plants poses significant safety concerns for the environment and human health [2]. Among radioactive wastes, one of the major concerns is the volatile radioactive iodine isotopes, including ^129^I and ^131^I [3]. The short-lived ^131^I (8.02 days) can directly interfere with human metabolism and cause serious health problems, including thyroid cancer [4]. On the other hand, the long-lived ^129^I (with a half-life of 1.57 × 10^7^ years) can contaminate the atmosphere and water resources for an extended period, adversely affecting the ecological environment and human health [5]. In this sense, there is an urgent to develop effective adsorbent materials for capturing radioiodine from vapor and solution phases of nuclear power waste.

Due to their high iodine removal efficiency and low operation costs, porous solid adsorbents have been seen as promising materials to capture and store radioiodines [6]. Common adsorbents have been extensively investigated in iodine removal, including zeolites [7], mesoporous silica [8], aerogels [9], and porous carbons [10]. Solid adsorbents containing silver (Ag) show better radioiodine removal performance compared to traditional liquid scrubbing techniques [11]. However, the adsorption capacities of these materials are limited and difficulties in the regeneration process restrict their usage in large-scale applications. In recent years, metal-organic frameworks (MOFs) have also been tested as solid adsorbents for iodine removal [12,13]. However, they exhibit low uptake capacities and poor physicochemical stability, making them unsuitable for industrial conditions. On the other hand, porous organic polymers (POPs) have been seen as promising materials for many applications, including separation, catalysis, and sensing, due to their unique properties including high surface area, tunable porosity, chemical versatility, and high physicochemical stability [14–22]. To date, several POPs have been reported for iodine capture and storage, including the subclasses of porous aromatic frameworks (PAFs) [23], conjugated microporous polymers (CMPs) [24], hyper-crosslinked polymers (HCPs) [25], covalent triazine frameworks (CTFs) [26], and covalent organic frameworks (COFs) [27,28]. In particular, COFs exhibit highly ordered and uniform pore structures, large surface areas, and accessible binding sites, making them ideal adsorbents for iodine removal [29]. Although recent studies have shown that COFs have remarkable iodine uptake capacities with fast adsorption kinetics, their low stability in thermal and acidic environments needs to be addressed for their practical applications.

Linkage chemistry plays a crucial role in determining both the crystallinity and stability of COFs. The reversibility of the linkage chemistry allows for dynamic error correction, facilitating the synthesis of thermodynamically stable and crystalline COFs during polymerization. However, this reversibility also results in low physicochemical stability [30,31]. While imine-based covalent linkages formed through Schiff-base chemistry result in crystalline COFs with strong covalent bonds, they are still susceptible to hydrolysis under harsh acidic and basic conditions [32,33]. Substituting these linkers with *β*-ketoenamine moieties significantly enhances the physicochemical stability of the COFs through intramolecular hydrogen bonding [34,35]. Nonetheless, the poor reversibility of *β*-ketoenamine linkages leads to low crystallinity and porosity [36]. Some alternative synthesis approaches have been reported to yield long-range ordered *β*-ketoenamine-linked COFs [37]. One such method was reported by Perepichka and colleagues, providing polymeric frameworks using the Michael addition-elimination reaction of *β*-ketoenols with amines [38]. This method offers a way to prepare crystalline and conjugated polymeric frameworks with exceptional hydrolytic stability. Despite their high physicochemical stability, there are limited studies on the usage of *β*-ketoenamine-linked COFs for capturing iodine in the literature [39–41].

Therefore, in this study, a *β*-ketoenamine-linked covalent organic framework (bCOF) is presented as an efficient adsorbent for the capture of iodine from vapor and organic solution. The bCOF was synthesized by modifying the method reported in the literature using *β*-ketoenol (**1**) and p-phenylenediamine (**2**) under Michael addition-elimination reaction conditions [38]. The bCOF has impressive iodine vapor uptake capacity of up to 2.51 g g^−1^ at 75 °C under ambient pressure due to its heteroatom-rich structure and high porosity. Furthermore, after five consecutive adsorption-desorption cycles, the bCOF exhibits significant reusability, retaining 83% of its initial iodine capture capacity. Several ex situ structural characterization methods were used to study the adsorption mechanism; the results of these mechanistic investigations demonstrated the formation of a charge-transfer complex between the electron-rich skeleton of the bCOF and adsorbed iodine species. Additionally, the bCOF has excellent iodine adsorption performance of up to 512 mg g^−1^ from a solution of iodine in cyclohexane. These findings demonstrate that the bCOF is a promising material due to its outstanding performance in adsorbing iodine from both vapor and solution.

## 2. Experimental

### 2.1. General

All chemicals and solvents were purchased from Sigma Aldrich (St. Louis, MO, USA), abcr (Karlsruhe, Germany), or Carlo Erba (Milan, Italy) and used without further purification unless otherwise stated. Analytical thin-layer chromatography (TLC) was performed using Merck (Darmstadt, Germany) alumina/silica gel 60-F254. The synthesized materials were purified by flash chromatography using Merck 70-230 mesh silica gel. The compound *β*-ketoenol (**1**) was synthesized according to the reported literature [38]. Nuclear magnetic resonance (NMR) analyses were carried out using a Varian (Palo Alto, CA, USA) 400 MHz spectrometer with ^1^H NMR at 400 MHz and ^13^C NMR at 100 MHz. Philips (Amsterdam, the Netherlands) X’Pert Pro and Bruker (Billerica, MA, USA) D8 Advance powder X-ray diffractometers were used to collect the powder X-ray diffraction (PXRD) patterns. Ultraviolet-visible (UV-Vis) spectra were collected using a Shimadzu (Tokyo, Japan) UV-2600i spectrophotometer. Thermogravimetric analysis (TGA) was performed using the PerkinElmer (Waltham, MA, USA) Thermogravimetric/Differential Thermal Analysis (TG/DTA) System under a nitrogen atmosphere at a rate of 10 °C/min until 800 °C. The Micromeritics (Norcross, GA, USA) ASAP 2020 surface area and porosity analyzer was used to collect nitrogen sorption isotherms at 77 K. The samples underwent preactivation through solvent exchange with immersion in THF and acetone for 48 h with the solvent refreshed every 8 h. Subsequently, the preactivated samples were degassed under reduced pressure at 100 °C for 16 h prior to gas adsorption analysis. Scanning electron microscopy (SEM) images of the bCOF and bCOF@I_2_ were taken at 8 kV using a Zeiss (Jena, Germany) EVO10. EDS mappings were also collected using a Zeiss EVO10 at 20 kV. Fourier transform infrared (FTIR) spectra were collected using a PerkinElmer 65 FTIR spectrometer. Raman spectroscopy analyses were conducted under ambient conditions using a Raman microscope (XploRA PLUS, Horiba, Kyoto, Japan) with operating wavelength of 785 nm and spectral resolution of 1.0 cm^−1^.

### 2.2. Synthesis of bCOF

Synthesis of the bCOF was performed following the procedure outlined in the literature, including some modifications [38]. A Pyrex tube was charged with *β*-ketoenol (**1**) (28.8 mg, 0.10 mmol), p-phenylenediamine (**2**) (16.2 mg, 0.15 mmol), 1,4-dioxane (2 mL), and mesitylene (2 mL). The mixture was sonicated and 6 M aqueous solution of acetic acid (0.5 mL) was added. The reaction mixture was then heated to 130 °C for 3 days in a flame-sealed tube. After the reaction was completed, the precipitate was first washed with water and 1,4-dioxane. Subsequently, the resulting solid was further washed with THF and acetone, respectively. The final solid was dried under a dynamic vacuum at 100 °C for 6 h, yielding the bCOF (31.3 mg, 79%).

### 2.3. Iodine capture (vapor)

To study gravimetric iodine vapor capture, 10 mg of bCOF was placed in a small vial. This vial was then placed in a sealed bottle that contained excess elemental iodine and an empty reference vial. The setup was heated to 75 °C in an oven under ambient pressure. After cooling to room temperature each time, the vial was weighed at different time intervals. The total vapor iodine uptake capacity was calculated using Eq. (1):


(1) $$Q_{\text{vapor}}=\frac{m_{2}-m_{1}}{m_{1}}$$

Here, *m_1_* and *m_2_* are the masses of the bCOF before and after iodine capture, respectively.

To test the recyclability of the bCOF, 5.0 mg of bCOF@I_2_ (iodine-loaded bCOF) was heated to 120 °C for 3 h under ambient pressure. The iodine release was measured during thermal treatment by weighing the sample at different time intervals. The iodine-loaded samples were also regenerated by immersing them in ethanol. bCOF@I_2_ (5.0 mg) was immersed in 5 mL of ethanol solution for 60 min and the desorption of iodine was followed by UV-Vis spectroscopy for the diluted samples. The recovered bCOF was dried at 100 °C under a dynamic vacuum. The regenerated sample was subjected to the gravimetric iodine vapor capture procedure for five consecutive cycles to test its recycling performance.

### 2.4. Iodine adsorption in cyclohexane solution

The iodine capture performance of the bCOF in an organic solvent was investigated using iodine solutions in cyclohexane and measured by collecting UV-Vis spectra. The bCOF (6.0 mg) was placed in a vial that was filled with 5 mL of a 250 mg L^−1^ iodine solution in cyclohexane. The UV-Vis spectra of the sample were collected at different time intervals to measure the iodine uptake capacity of the bCOF in cyclohexane. A standard calibration curve of iodine in cyclohexane was prepared using solutions of 1000, 900, 800, 600, 500, 400, 200, 100, and 50 mg L^−1^ concentrations. To determine the maximum iodine uptake capacity of the bCOF in cyclohexane solutions of 1000, 800, 400, and 250 mg L^−1^ concentrations, 6.0 mg of bCOF was immersed in each solution without shaking and UV-Vis spectra were collected after 20 h.

### 2.5. Kinetic studies

The adsorption kinetics of the bCOF in cyclohexane solution were calculated with nonlinear pseudo-first-order and nonlinear pseudo-second-order models, as shown in Eqs. (2) and (3), respectively:


(2) $$Q_{\text{t}}=Q_{\text{e}}(1-e^{-k_{1}t})$$

(3) $$Q_{\text{t}}=\frac{\left(k_{2}Q_{{\text{e}}^{2}}t\right)}{\left(1+k_{2}Q_{\text{e}}t\right)}$$

Here, *Q_t_* and *Q_e_* are the amounts of adsorbed iodine at time *t* and after reaching equilibrium, respectively. *k_1_* and *k_2_* denote pseudo-first-order and pseudo-second-order rate constants, respectively.

Langmuir (Eq. (4)) and Freundlich (Eq. (5)) adsorption isotherm models were used to understand the adsorption behavior of the bCOF. The models were as follows:


(4) $$Q_{\text{e}}=\frac{\left(Q_{\text{max}}K_{\text{L}}C_{\text{e}}\right)}{\left(1+K_{\text{L}}C_{\text{e}}\right)}$$

(5) $$Q_{\text{e}}=K_{\text{F}}(Q_{{\text{e}}^{1/\text{n}}})$$

Here, *Q_e_* and *C_e_* are the adsorbed amount of iodine in equilibrium and iodine concentration, respectively. *K_L_* and *K_F_* are the adsorption constants of the Langmuir and Freundlich models. *n* and *Q_max_* denote the Freundlich linearity index and theoretical maximum adsorption value, respectively.

## 3. Results and discussion

**Figure 1:**
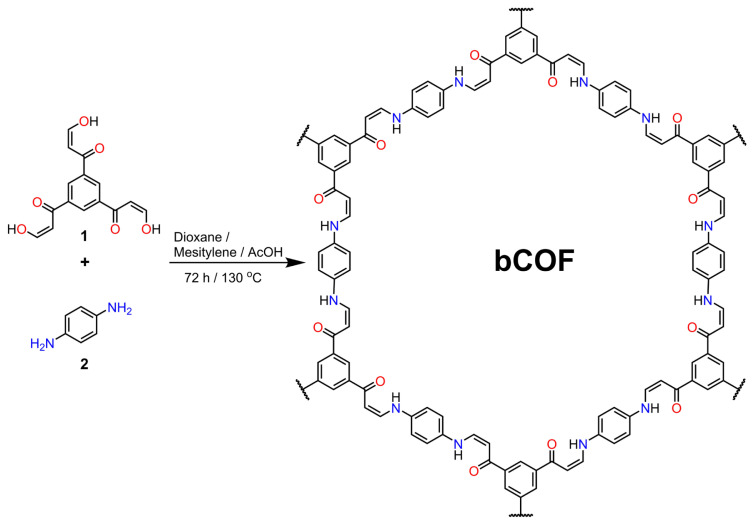
Schematic representation of the pathway for the synthesis of the bCOF.

The bCOF was synthesized by modifying the reported literature method using *β*-ketoenol (**1**) and p-phenylenediamine (**2**) through the Michael addition-elimination reaction (Figure 1) [38]. The reaction was carried out in a flame-sealed ampoule at 130 °C in the presence of an aqueous acetic acid solution as a catalyst.

**Figure 2:**
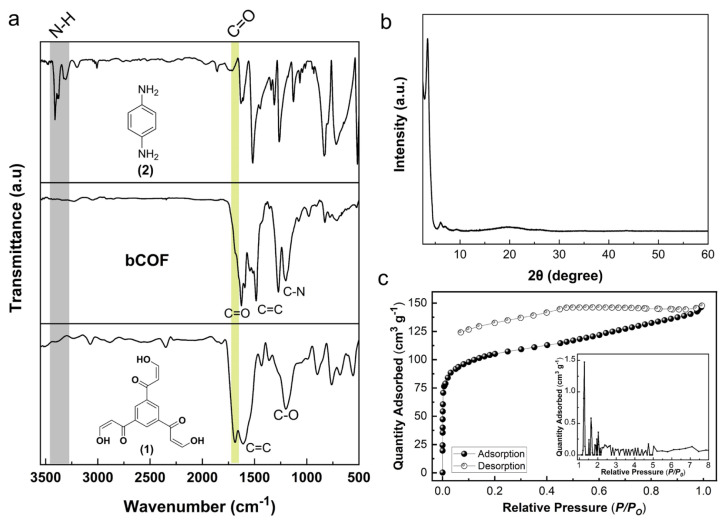
(a) FTIR spectra of bCOF, *β*-ketoenol (**1**), and *p*-phenylenediamine (**2**). (b) PXRD pattern of bCOF. (c) N_2_ adsorption-desorption isotherm of bCOF collected at 77 K, with the inset showing pore size distribution of bCOF calculated using the N_2_ adsorption isotherm.

As depicted in Figure 2a, the synthesis of the bCOF was verified using FTIR analysis. The FTIR spectrum of the bCOF showed distinct peaks at 1625 and 1200 cm^−1^, corresponding to the C=O and C-N functional groups, respectively. The successful condensation of *β*-ketoenol (**1**) and p-phenylenediamine (**2**) groups into *β*-ketoenamine linkages was evidenced by the disappearance of the stretching bands of N-H and C-O at about 3420 and 1210 cm^−1^, respectively.

The X-ray diffraction (XRD) pattern of the bCOF is fully consistent with the previously reported COF structure (Figure 2b) [38]. The bCOF revealed four distinct reflections at 2θ values of 3.50°, 6.10°, 7.05°, and 9.40°. The reflection at 3.50° can be attributed to the (100) plane. The observed weak and broad diffraction peak at 2θ = 26° corresponds to the (001) reflection, which can be attributed to stacking layers of the bCOF with vertical spacing of 3.4 Å. The porosity of the bCOF was investigated using nitrogen adsorption-desorption isotherms collected at 77 K (Figure 2c). The steep increase of N_2_ uptake at low pressures was associated with the presence of micropores in the bCOF structure. The bCOF demonstrates a combination of type I and type II adsorption isotherms, indicating the presence of both micro- and mesopores. The observed H4 and broad hysteresis loop over a wide pressure range also verify the presence of both micro- and mesopores, suggesting the existence of defects due to the low crystallinity of the bCOF [15]. The BET surface area of the bCOF was calculated as 388 m^2^ g^−1^. The surface area of the bCOF is smaller compared to previously published similar COF structures, which can be attributed to the presence of defects arising from the low crystallinity of the bCOF [37,38]. The pore size distribution of the bCOF was determined from the nitrogen adsorption isotherm using nonlocal density functional theory (NLDFT). As shown in the inset of Figure 2c, the micropore region dominates the pore size distribution, which indicates the microporous nature of the bCOF with total pore volume of 0.23 cm^3^ g^−1^.

**Figure 3:**
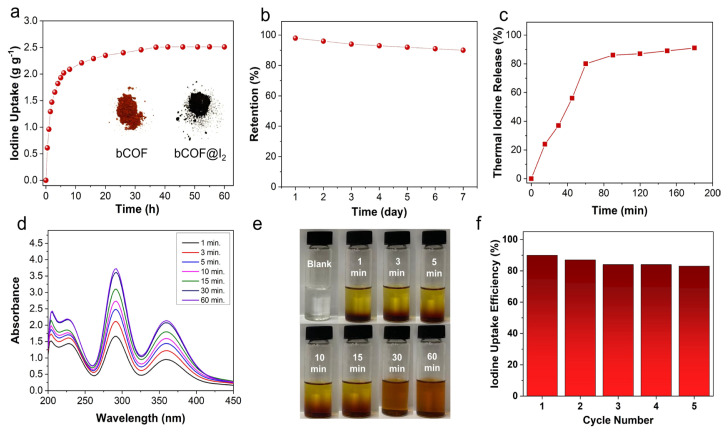
Iodine vapor uptake and recyclability of bCOF: (a) Gravimetric iodine vapor adsorption of bCOF as a function of time at 75 °C under ambient pressure (inset shows the color change of bCOF upon exposure to iodine vapor). (b) Retention performance of iodine-loaded bCOF (bCOF@I_2_) at 25 °C and ambient pressure for 7 days. (c) Thermal release efficiency of bCOF@I_2_ by heating at 120 °C for 3 h. (d) Time-dependent UV-Vis spectra of iodine release of bCOF@I_2_ in ethanol. (e) Digital images of iodine release from bCOF@I_2_ after immersion in ethanol show the color of the solution changing from clear to dark brown over time. (f) Recyclability performance of bCOF for five adsorption-desorption consecutive cycles.

The bCOF’s high surface area, large pore volume, heteroatom, and electron-rich skeleton make it a promising platform for capturing radioactive iodine vapor. A gravimetric measurement was conducted under ambient pressure in a closed static system to explore its iodine vapor capture performance. The bCOF was placed in a sealed bottle containing saturated iodine vapor at 75 °C under ambient pressure. The iodine uptake performance was calculated by measuring the weight increase at different time intervals. As depicted in Figure 3a, the bCOF exhibited rapid iodine vapor adsorption in the first 12 h and reached adsorption saturation within 36 h. The color of the bCOF changed from dark red to black after it was exposed to iodine vapor, as illustrated in the inset of Figure 3a. The maximum iodine vapor uptake capacity of the bCOF was measured as 2.51 g g^−1^. The iodine uptake performance of the bCOF was compared with various porous adsorbents as summarized in the Table. Although the bCOF shows slightly lower iodine vapor uptake capacity compared to some recently reported COF-based adsorbents [27,28,29,40], it still delivers reasonably high or similar uptake performance compared to several porous materials tested in iodine capture (Table), including porous carbons (NT-POP@800-1 = 0.68 g g^−1^ [42], AC = 2.42 g g^−1^ [43]), MOFs (UiO-66 = 0.680 g g^−1^ [44], HKUST-1 = 0.375 g g^−1^ [45]), and POPs (BPTz-POP = 2.16 g g^−1^ [16], PAF-25 = 2.60 g g^−1^ [23], HCMP =1.59 g g^−1^ [24], NRPP-1 = 1.92 g g^−1^ [46], PAN-FPP5 = 2.22 g g^−1^ [47], PTZ-TPC-MA= 1.98 g g^−1^ [48]).

Gravimetric measurement data were also used to investigate iodine vapor capture adsorption kinetics. The average adsorption rate for 80% of total adsorption (K_80%_) was used to determine the adsorption kinetics, which is a convenient parameter when testing the practical performance of adsorbents. The K_80%_ value of the bCOF was found to be 0.37 g h^−1^, indicating the high iodine adsorption kinetics of the bCOF compared to reported adsorbents with similar or higher iodine uptake capacity than bCOF, such as COF-TAPB (0.33 g g^−1^ h^−1^ [49]), HCP-Cl (0.04 g g^−1^ h^−1^ [50]), COF-OH-50 (0.189 gg^−1^ h^−1^ [51]), and TpPa-SO_3_K (0.33 g g^−1^ h^−1^ [52]). The iodine-loaded sample (bCOF@I_2_) was also tested for iodine retention performance under ambient conditions. bCOF@I_2_ effectively retained iodine for a period of 7 days without noticeable iodine leakage under ambient conditions (Figure 3b).

**Figure 4:**
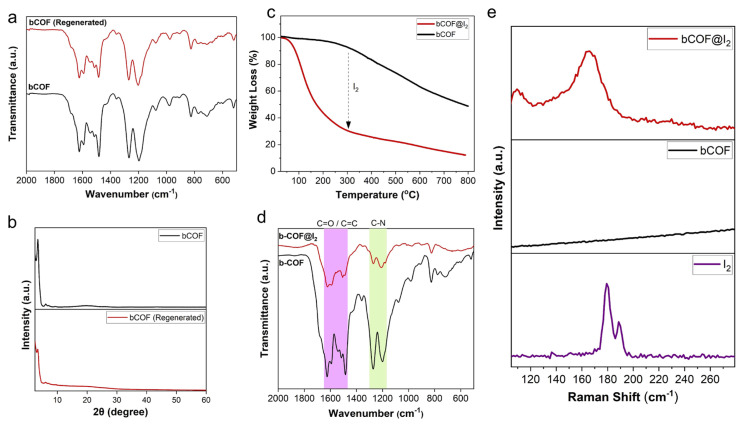
Comparison of the FTIR spectra (a) and PXRD patterns (b) of bCOF and regenerated bCOF. Investigation of I_2_/bCOF interaction: (c) TGA curves of bCOF and iodine-loaded bCOF@I_2_ under N_2_ atmosphere in the temperature range of 25–800 °C at heating rate of 10 °C min^−1^. (d) FTIR spectra of bCOF and bCOF@I_2_. (e) Raman spectra of I_2_, bCOF, and bCOF@I_2_.

In order to test the recyclability of the bCOF, the iodine-loaded sample (bCOF@I_2_) was heated to 120 °C for 3 h under ambient pressure. Iodine release was measured using gravimetric techniques. As shown in Figure 3c, the majority of captured iodine was released within 1 h, with equilibrium reached in 3 h with thermal release efficiency of 89%. Iodine-loaded samples (bCOF@I_2_) were also soaked in ethanol to extract the adsorbed iodine. The release was followed by UV-Vis spectroscopy by collecting the spectra at different time intervals (Figure 3d). The observed peaks at 291 and 350 nm corresponded to the presence of polyiodide species. Notably, the color of the ethanol solution changed immediately from colorless to dark yellow after immersing bCOF@I_2_ into the solution (Figure 3e). The captured iodine was released quickly within 10 min and equilibrium was reached after 30 min. These regenerated samples were then dried in a dynamic vacuum at 100 °C before being reused in iodine vapor uptake measurements. The regenerated bCOF showed high uptake capacity of up to 83% of its initial performance after five consecutive adsorption-desorption cycles (Figure 3f). The structural integrity of the regenerated samples was verified through FTIR and PXRD analyses. The FTIR spectra of pristine and regenerated bCOF displayed identical IR bands, as shown in Figure 4a, indicating that the bCOF retained its chemical structural integrity following the regeneration process. While the regenerated sample displayed a PXRD pattern comparable to that of the pristine bCOF, the intensity of peaks of the bCOF was slightly attenuated, suggesting some structural deformation and a decrease in crystallinity due to the regeneration process, as shown in Figure 4b [14,53]. These findings align with the observed capacity loss of the bCOF over multiple cycles.

To investigate the iodine adsorption mechanism and elucidate the interaction between the bCOF and I_2_, a series of analytical techniques, including TGA, FTIR, and Raman spectroscopy, were carried out. TGA was used to evaluate the thermal stability of the bCOF and the regeneration of bCOFs from an iodine-saturated sample (I_2_@bCOF) through thermal treatment (Figure 4c). The pristine bCOF showed high thermal stability at up to 350 °C without significant weight loss. On the other hand, the TGA curve of bCOF@I_2_ displayed a mass loss of about 63% between 100 °C and 300 °C due to the release of captured iodine. The estimated captured iodine weight loss for the bCOF was calculated as 71.5% based on gravimetric measurements. The difference in mass loss observed between TGA and gravimetric measurements suggests a significant interaction between the bCOF and iodine molecules, potentially leading to the entrapment of some iodine molecules within the pores of the bCOF. The thermal treatment may also cause pore collapse or blockage, ultimately resulting in the trapping of iodine molecules. The TGA results were consistent with the thermal release experiment, both indicating an incomplete removal of iodine [39,54].

As shown in Figure 4d, the bCOF FTIR spectroscopy results exhibited noticeable changes after the iodine vapor treatment. After the adsorption of iodine, the stretching vibrations of C=O at 1625 cm^−1^, C=C 1590 cm^−1^, and C-N at 1200 cm^−1^ decreased in intensity and shifted in the bCOF@I_2_ compared to the pristine bCOF. This notable change in intensity and shift for the FTIR bands indicates a strong interaction between all functional units of the bCOF and adsorbed iodine species [14,24]. It highlights the crucial role of the conjugated heteroatom-rich skeleton of the bCOF in the adsorption of iodine vapor. Raman spectroscopy was carried out to understand the chemical nature of adsorbed iodine species in bCOF@I_2_. As depicted in Figure 4e, the peaks that appeared at 109 and 166 cm^−1^ can be attributed to the stretching vibration of I_3_^−^ and the characteristic signal of I_5_^−^, respectively. As observed in previous studies, the formation of polyiodide species (I_3_^−^ and I_5_^−^) indicates the formation of charge transfer complexes between adsorbed iodine and the electron-rich skeleton [4,6,29,39,52]. These strong charge transfer interactions make the bCOF an effective adsorbent for capturing iodine.

**Figure 5:**
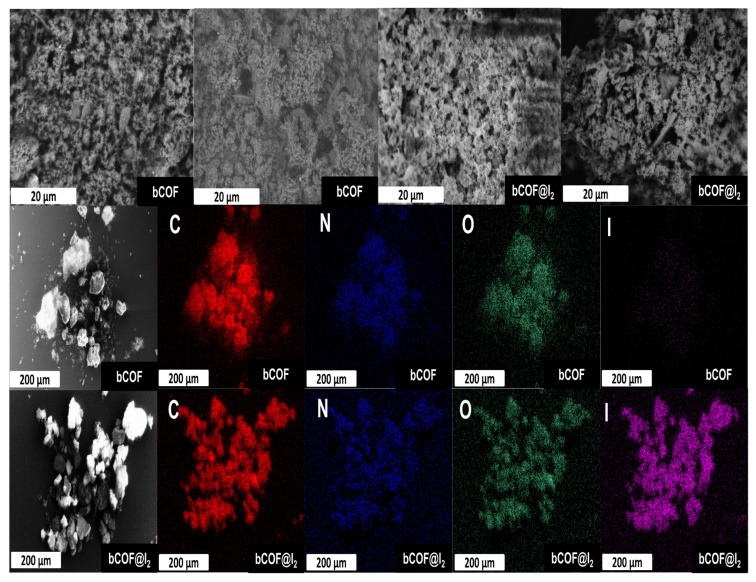
SEM images and EDX elemental maps of bCOF and bCOF@I_2_ for carbon (C), nitrogen (N), oxygen (O), and iodine (I).

As shown in Figure 5, SEM images of the bCOF after iodine uptake revealed no morphological changes, implying that iodine accommodation occurred in the pores of the bCOF [54]. The iodine capture properties of the bCOF were also investigated using energy-dispersive X-ray (EDX) analysis. The EDX elemental mapping of bCOF@I_2_ demonstrated uniformly distributed iodine within the pores of the bCOF (Figure 5).

**Figure 6:**
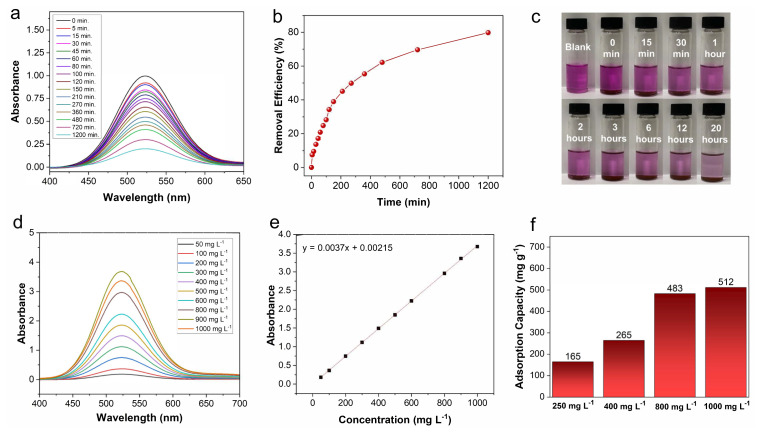
(a) Time-dependent UV-Vis spectra of iodine solution of 250 mg L^−1^ in cyclohexane after adding bCOF. (b) Iodine uptake efficiency of bCOF from iodine solution in cyclohexane (250 mg L^−1^). (c) Optical images for iodine uptake efficiency of bCOF from the solution of iodine-cyclohexane. d) UV-Vis spectra of cyclohexane solution of iodine at different concentrations. (e) Fitting curve of iodine-cyclohexane solution based on absorbance at 526 nm with an R-square value of 0.9999. (f) Maximum iodine uptake capacity of bCOF from iodine solutions in cyclohexane with concentrations of 250, 400, 800, and 1000 mg L^−1^.

The iodine capture performance of the bCOF from an organic solution was investigated using a solution of iodine in cyclohexane. As shown in the time-dependent UV/Vis absorption spectra of the bCOF (Figure 6a), the absorption intensity of the cyclohexane-iodine solution (250 mg L^−1^) was rapidly decreased in the first 6 h and slowly continued to decrease until it reached removal efficacy of 80% in 20 h (Figure 6b). As observed in Figure 6c, the color of the iodine solution in cyclohexane changed from purple to colorless in 20 h after the addition of the bCOF.

UV-Vis spectra of the cyclohexane solution of iodine at various concentrations were obtained (Figure 6d), and the fitting curve of the iodine-cyclohexane solution was calculated based on the absorbance at 526 nm with an R-square value of 0.9999 (Figure 6e), which was used to determine the iodine uptake capacities of the bCOF. The uptake capacity of the bCOF increased with increasing concentrations of iodine in cyclohexane. The maximum iodine capacity of the bCOF in cyclohexane reached up to 165, 265, 483, and 512 mg g^−1^ from a solution of iodine in cyclohexane of 250, 400, 800, and 1000 mg L^−1^, respectively (Figure 6f). These values were comparable or even higher than those of most reported adsorbents, like TPFM (292 mg g^−1^ iodine uptake capacity in 1000 mg L^−1^) [55], P-TzTz (494 mg g^−1^ iodine uptake capacity in 1000 mg L^−1^) [56], and CD-POP (297 mg g^−1^ iodine uptake capacity in 500 mg L^−1^) [57].

**Figure 7:**
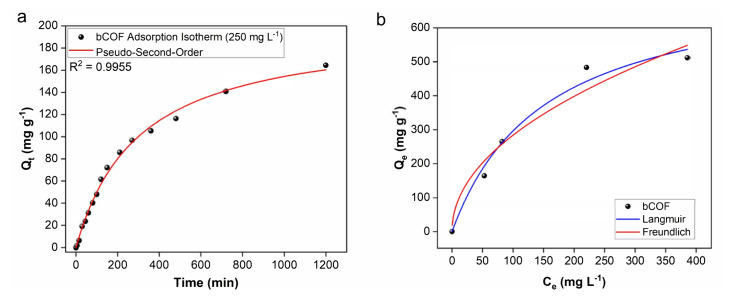
(a) Pseudo-second-order model of bCOF (solution of iodine in cyclohexane with a concentration of 250 mg L^−1^). (b) Langmuir and Freundlich models for the iodine adsorption of bCOF in cyclohexane solution.

The adsorption kinetics of the bCOF were also explored through pseudo-first-order and pseudo-second-order models. The bCOF adsorption values from the cyclohexane solution of 250 mg L^−1^ showed good fits with the pseudo-second-order kinetic model (R-square value of 0.9955) compared to the pseudo-first-order kinetic (R-square value of 0.9883), indicating that the absorption kinetics of the bCOF are based on the chemisorption process (Figure 7a) [58]. Langmuir and Freundlich models were also used to understand the adsorption mechanism of the bCOF from iodine-cyclohexane solutions. The correlation coefficient for Langmuir and Freundlich isotherms was found to be 0.9836 and 0.9115, respectively (Figure 7b). Thus, the adsorption fit better with the Langmuir model, suggesting a monolayer adsorption process [59].

## 4. Conclusion

In this study, a *β*-ketoenamine-linked covalent organic framework (bCOF) was synthesized through the Michael addition-elimination reaction. Its high porosity and heteroatom-rich conjugated backbone make it a promising platform to capture iodine from both vapor and solution. It shows moderate iodine vapor adsorption performance with uptake capacity of 2.51 g g^−1^ at 75 °C under ambient pressure. Due to the physiochemical stability of the *β*-ketoenamine linkage, the bCOF revealed high recyclability for up to five consecutive iodine vapor adsorption-desorption cycles with 17% loss from its initial performance. Ex situ analysis was also carried out to study the adsorption mechanism. The mechanistic studies highlighted the strong charge transfer interaction between adsorbed iodine species and the electron-rich skeleton of the bCOF. Furthermore, the bCOF exhibited exceptional capability for adsorbing iodine from a cyclohexane-iodine solution with maximum adsorption capacity of up to 512 mg g^−1^. These findings suggest that the bCOF is a highly promising material due to its exceptional ability to adsorb iodine from both vapor and solution.

## Figures and Tables

**Table:** Comparison of the iodine vapor capture performance of bCOF with recently reported porous materials.

Entry	Adsorbents	Temperature (°C)	Iodine uptake capacity (g g^−1^)	Reference

1	SCU-COF-2	75	6.0	[14]

2	FcTZ-POP	75	3.96	[16]
	BPTz-POP		2.16	

3	PAF-23	75	2.71	[23]
	PAF-24		2.76	
	PAF-25		2.60	

4	HCMP-1	85	1.59	[24]
	HCMP-2		2.81	
	HCMP-3		3.16	
	HCMP-4		2.22	

5	TPB-DMTP COF	77	6.26	[27]
	TTA-TTB COF		4.95	

6	COF-Ph	77	4.47	[28]

7	USTB-1	75	4.45	[29]
	USTB-1c		5.80	

8	COF-TpgDB		2.75	[40]
	COF-TpgBD		1.81	
	COF-TpgTd		1.66	

9	NT-POP@800-1	77	0.68	[42]

10	AC	77	2.42	[43]

11	UiO-66	75	0.68	[44]

12	HKUST-1	75	0.38	[45]

13	NRPP-1	80	1.92	[46]

14	PAN-FPP5	72	2.22	[47]

15	PTZ-TPC-MA	75	1.98	[48]

16	bCOF	75	2.51	This work
